# Can Machine Learning Assist in Diagnosis of Primary Immune Thrombocytopenia? A Feasibility Study

**DOI:** 10.3390/diagnostics14131352

**Published:** 2024-06-26

**Authors:** Haroon Miah, Dimitrios Kollias, Giacinto Luca Pedone, Drew Provan, Frederick Chen

**Affiliations:** 1 Centre of Immunobiology, Blizard Institute, Queen Mary University of London, London E1 2AT, UK; haroonmiah@qmul.ac.uk (H.M.); d.provan@nhs.net (D.P.); 2 Haematology Department, Barts Health NHS Trust, London E1 1BB, UK; giancinto.pedone@nhs.net; 3 School of Electronic Engineering & Computer Science, Queen Mary University of London, London E1 4NS, UK

**Keywords:** primary immune thrombocytopenia, machine learning, artificial intelligence, healthcare, diagnosis, blood tests, model fairness, feature importance, explainability, UK adult ITP registry

## Abstract

Primary Immune Thrombocytopenia (ITP) is a rare autoimmune disease characterised by the immune-mediated destruction of peripheral blood platelets in patients leading to low platelet counts and bleeding. The diagnosis and effective management of ITP are challenging because there is no established test to confirm the disease and no biomarker with which one can predict the response to treatment and outcome. In this work, we conduct a feasibility study to check if machine learning can be applied effectively for the diagnosis of ITP using routine blood tests and demographic data in a non-acute outpatient setting. Various ML models, including Logistic Regression, Support Vector Machine, k-Nearest Neighbor, Decision Tree and Random Forest, were applied to data from the UK Adult ITP Registry and a general haematology clinic. Two different approaches were investigated: a demographic-unaware and a demographic-aware one. We conduct extensive experiments to evaluate the predictive performance of these models and approaches, as well as their bias. The results revealed that Decision Tree and Random Forest models were both superior and fair, achieving nearly perfect predictive and fairness scores, with platelet count identified as the most significant variable. Models not provided with demographic information performed better in terms of predictive accuracy but showed lower fairness scores, illustrating a trade-off between predictive performance and fairness.

## 1. Introduction

Primary Immune Thrombocytopenia (ITP) is an autoimmune disease characterised by the immune-mediated destruction of peripheral blood platelets in patients leading to low platelet counts and bleeding [1]. ITP affects approximately 6.4 per 100,000 people and although life-threatening bleeds are relatively rare, it can lead to catastrophic intracranial bleeding and death [2].

The diagnosis and effective management of ITP is challenging because there is no established test to confirm the disease and no biomarker with which one can predict the response to treatment and outcome. Currently, ITP diagnosis is made by exclusion of other causes of low platelet counts, and management decisions rely heavily on clinical judgment. The diagnosis therefore relies on blood tests to demonstrate a low platelet count and tests that exclude other conditions.

Artificial Intelligence (AI) encompasses the development of computer programs designed to simulate human intelligence. These programs operate on a complex framework of algorithms enabling machines to emulate human cognitive functions such as learning and problem-solving. A prominent subfield of AI, Machine Learning (ML), leverages vast datasets to identify patterns and generate predictions. What makes these algorithms unique lies in their capacity to concurrently process both linear and nonlinear variables, facilitating the recognition of intricate patterns. This capability significantly enhances their accuracy in making predictions, thus broadening their applicability across various complex scenarios. Although ML has previously been used in healthcare to automate hospital systems, recently, it has also been utilised in the diagnosis, early detection, and monitoring of diseases [3,4,5]. In recent years, there have been several successful applications of AI in various medical conditions, such as the diagnosis of LA fibrillation and evaluation of prognosis in COVID-19 [6,7,8].

The potential impact of employing an ML system to predict ITP in patients using routine blood tests and demographic information includes streamlining clinical pathways, facilitating rapid referral, improving patient safety and outcomes, as well as improving efficiency.

Currently, the diagnosis of ITP often involves multiple healthcare visits and tests, which can be time-consuming and stressful for patients. The pathway typically involves initial suspicion by a GP, referral to a general clinic, and subsequent referral to a specialist; if ITP is suspected, each step may require separate appointments and assessments. Therefore, an ML system could identify patterns or anomalies that suggest ITP enabling GPs or community clinics to make informed decisions about the necessity of specialist referrals without the need for initial physical assessments by a haematologist. This streamlined approach means that patients suspected of having ITP could bypass certain steps in the traditional pathway, reducing the time for diagnosis and treatment. Early and accurate predictions can minimise unnecessary tests and procedures, thus reducing healthcare costs and patient burden. Additionally, by incorporating ML predictions, community clinics can quickly identify and refer high-risk patients directly to specialist haematologists. This direct referral process avoids delays that occur when waiting for multiple consultations and non-specialist assessments. ML models, trained on large datasets, may potentially recognise subtle patterns in blood tests that are not immediately obvious to human clinicians. This could increase the accuracy of initial assessments in community clinics, ensuring that referrals to specialists are well-founded and necessary.

Furthermore, the early detection and treatment of ITP are crucial to prevent complications such as severe bleeding. ML systems can operate continuously, analysing incoming data from routine blood tests performed for other reasons, thus potentially identifying ITP cases that might otherwise go unnoticed until symptoms worsen. ML can further help in personalising the patient care pathway. For instance, by analysing demographic and medical history alongside test results, ML may predict the severity of ITP or suggest the most effective treatments based on similar cases. Finally, hospitals and clinics can better allocate their resources, including specialist time and hospital beds, by ensuring only patients with a high likelihood of having ITP are referred for specialist care. This can lead to a more efficient use of healthcare resources. Reducing the number of steps in the patient journey not only improves the patient experience but also reduces healthcare costs associated with multiple clinic visits and unnecessary testing.

In this work, we conduct a feasibility study to check if ML can be applied effectively for diagnosis of ITP in a non-acute outpatient setting by using simple but widely available blood test results. In other words, we assess if an ML model that takes as input blood test results can effectively distinguish between ITP and non-ITP patients. We utilise various widely used ML models, namely Logistic Regression (LogR), Support Vector Machine (SVM), k-Nearest Neighbor (k-NN), Decision Tree (DT) and Random Forest (RF). For each model, we investigate two approaches—the demographic-unaware and the demographic-aware ones. Regarding the former, we only provide as input to the model the blood test results, whereas for the latter (apart from the blood test results) we further provide as input to the model the patients’ demographics (age, race and gender).

The blood tests that we use include the routine full blood count that incorporates the platelet count, haemoglobin, red cell indices, and the differential white cell count, and biochemical screening consisting of liver and renal function tests. These are routine tests that all patients have, irrespective of suspected diagnosis when they attend a community-facing general outpatient clinic. Following diagnostic work-up, these patients are then referred on to specialist disease-specific clinics, such as our hospital’s haematology clinic for further confirmatory tests and clinical assessment. ITP is diagnosed based on a low platelet count (below $100\times 10^{9}$/L) and the exclusion of other causes of thrombocytopenia. Blood tests used routinely in outpatient clinics are able to identify low platelet counts and exclude most other causes of low platelets. In addition to identifying a low platelet count, the full blood count (FBC) informs of any abnormalities of the haemoglobin, white cell count, including neutrophil counts, and of red cell indices such as mean corpuscular volume (MCV). Common causes of low platelet counts such as deficiency in vitamin B12 or folic acid, myelodysplasia, lymphoproliferative disease, and bone marrow failure syndromes are associated with an abnormal FBC. An abnormal alanine aminotransferase (ALT), a commonly tested liver enzyme, and a measure of liver function, could indicate liver disease, another common cause of low platelets. In this study, we used the dataset from the UK Adult ITP Registry (UKITPR). Registries are established in order to collect sufficient patient data to study the natural history and outcomes of patients, in particular of patients with rare diseases where there is insufficient experience and data available in a single institution. ITP is a rare disease, and has therefore historically not been well-studied. For this reason, the UKITPR was established to collect patients’ demographic, clinical and laboratory data. The registry has recruited over 5000 patients from across the UK and from over 100 hospitals.

We conduct extensive experiments with all ML models to discover which model achieved the highest performance and which of the two previously mentioned approaches (i.e., demographic-aware or demographic-unaware) worked the best. Additionally, we conduct experiments to assess how biased each ML model is with regard to the patients’ demographics (age, race and gender). Finally, for interpretability of the achieved results, we conduct an analysis to understand the contribution of each input variable to the prediction performance of every utilised model. In this way, we can assess how important and influential each input variable is to every model. The following are the key insights from all our experiments. At first, the Random Forest and the Decision Tree ML models achieved the highest (and perfect) predictive performance across all ML models. An interesting observation is that they achieved the same performance in the two previously described approaches (demographic-aware and demographic-unaware ones). Secondly, these two models are the fairest among all other ML models and they are also considered fair (unlike the other ML models). Another interesting observation is that these two models achieved the same fairness score across the two approaches. Thirdly, we observe that the the low platelet count is the most important input variable that greatly (or solely) affects the decision-making of these models; a finding that is consistent with how ITP is diagnosed in the medical world (the low platelet count is also in the top-three most influential input variables for the rest of the ML models). Finally, across all studied ML models, the demographic-aware approach achieves worse performance (than the demographic-unaware one), whilst it is fairer.

## 2. Related Work

Several studies have used ML to predict pre-defined outcomes in selected populations of ITP. In [9], several ML models were tested for predicting the risk of critical bleeding in a large retrospective and prospective multicentre cohort of over 3000 ITP patients, using input variables such as demographic data, comorbidities, chronicity of ITP, drugs and platelet counts. Half the cohort, constituting the retrospective data, was used for training and internal validation, and the remaining prospective data was used for testing the performance of the ML models. The best-performing model in predicting critical bleeding as defined by the International Society of thrombosis and haemostasis in this study was Random Forest, which achieved an AUC score of 0.89; Random Forest was followed (in terms of achieved performance) by XGBoost, Light GM and Logistic Regression.

In [10], multivariate Logistic Regression was utilised to predict death within 30 days of an intracranial haemorrhage (ICH) in ITP patients. Similar to the work of [9], it used a multitude of variables based on demographics, comorbidities, platelet counts at set time points from diagnosis of ITP, and drugs. Multicentre data from 142 patients with ICH from ITP were used for training and testing ML models. The performance of their model in predicting mortality from intracranial haemorrhage was evaluated using ROC with AUC value for test cohort of 0.942.

Another study [11] explored the prediction of ITP chronicity in children. In this study, a cohort of 696 single-centre paediatric ITP patients were used and variables analysed at the time of ITP diagnosis were used to predict which patients will develop chronic ITP. Various ML models were tested and Random Forest was found to have the best performance in distinguishing chronic ITP from acute ITP, achieving an ROC AUC score of 0.8. In addition to demographic data and clinical features, more extensive blood tests were used in this study as variables for ML. These included presenting platelet count, immature platelet counts, platelet indices such as mean platelet volume (MPV), lymphocyte count, direct antiglobulin test (DAT) for immune haemolysis and antinuclear antibody (ANA). In a smaller study of 60 ITP patients [12], ITP relapse was predicted after cessation of steroids based on the profile microbiome of the gut. Using Random Forest, the study was able to predict relapse of ITP and response to thrombopoietin receptor agonists based on the characteristics of the microbiota, including the species of microbes. The ROC AUC values were 0.87 in distinguishing between relapse and remission of ITP.

## 3. Materials and Methods

### 3.1. Problem Statement

The dataset consists of *N* data points $(x_{i},y_{i}),$ j=1,…,N with $x_{i}\in R^{d}$ being the input variables and their corresponding target label $y_{i}\in \left\{0,1\right\}$ (i.e., binary classification). Moreover, each $x_{i}$ is associated with the sensitive variables $s_{i}=\langle s_{\mathrm{Age}},s_{\mathrm{Gender}},s_{\mathrm{Race}}\rangle$, where $s_{\mathrm{Age}}\in R$, $s_{\mathrm{Gender}}\in \left\{\mathrm{male},\;\mathrm{female}\right\}$ and $s_{\mathrm{Race}}\in \left\{\mathrm{White},\;\mathrm{Black},\;\mathrm{Asian},\;\mathrm{Other}\right\}$. A sensitive variable is a label which corresponds to a protected characteristic which we do not want to base a model’s decisions on. Lets us mention that we adopted a commonly accepted race classification from the U.S. Census Bureau and thus we defined the following four race groups (in alphabetical order): Asian, Black (or African American), White (or Caucasian) and Other (which includes Indian or Alaska Native, and Native Hawaiian or Other Pacific Islander). In other words, these sensitive variables are the subjects’ demographics. We develop and evaluate two approaches: a demographic-aware approach and a demographic-unaware approach. Their corresponding goals are to model $p(y_{i}|x_{i},s_{i})$ and $p(y_{i}|x_{i})$, respectively.

### 3.2. Machine Learning Models

Five classical Machine Learning (ML) models, namely Logistic Regression (LogR) [13], Support Vector Machine (SVM) [14], k-Nearest Neighbor (k-NN) [15], Decision Tree (DT) [16] and Random Forest (RF) [17], were developed to diagnose ITP.

LogR is a statistical method used for binary classification tasks in ML. It models the probability that a given input belongs to a particular class by employing the logistic function to map predicted values to probabilities between 0 and 1. In more detail, by modeling the log-odds of the binary outcome as a linear combination of predictor variables, Logistic Regression applies the logistic function to transform these log-odds into probabilities between 0 and 1. This approach allows Logistic Regression to handle cases where the relationship between the independent variables (i.e., the input) and the dependent variable (i.e., the output) is not strictly linear. This approach not only facilitates accurate classification (it is highly effective as it has robust performance on linearly separable data) but also enhances model interpretability: the coefficients provide clear insights into how each predictor influences the outcome. Each coefficient signifies the change in the log-odds of the dependent variable for a one-unit increase in the predictor, making it straightforward to understand and communicate the impact of different features. All these make LogR a popular choice for initial modelling and baseline comparisons in ML research.

SVM is a class of supervised learning algorithm that works by finding the optimal hyperplane that best separates data into different classes. This hyperplane is determined by maximizing the margin between the nearest data points of each class, known as support vectors, which are critical in defining the decision boundary. SVMs are particularly effective in high-dimensional spaces and are robust to overfitting, especially in scenarios where the number of dimensions exceeds the number of samples. The interpretability of SVMs stems from the explicit identification of support vectors and the derived coefficients of the hyperplane, which indicate the importance and contribution of each feature in the classification decision. Additionally, by utilizing kernel functions, SVMs can handle nonlinear classification tasks by transforming the input space into higher dimensions where a linear separation is feasible. The choice of kernel function (linear, polynomial, radial basis function, etc.) provides flexibility in modeling complex relationships between features, enhancing the algorithm’s applicability across various types of data.

The k-NN algorithm is a simple yet powerful non-parametric method that operates on the principle that the classification of a data point is determined by the majority class among its k closest neighbors in the feature space, where distance metrics such as Euclidean, Manhattan, or Minkowski are used to measure proximity. Its non-parametric nature means it does not make any assumptions about the underlying data distribution, making it highly flexible and applicable to a wide range of datasets. The interpretability of k-NN is straightforward: the prediction for a data point is directly influenced by its neighboring points, providing clear insights into the local structure of the data. Analysts can easily understand and visualize why a particular classification was made by examining the nearest neighbors. However, k-NN’s performance can be significantly affected by the choice of k and the distance metric, as well as being computationally intensive for large datasets due to the need to compute distances between the query instance and all training samples. Despite these challenges, k-NN remains a widely used baseline algorithm due to its simplicity and effectiveness.

DT is a versatile and widely used algorithm in machine learning, renowned for its simplicity and interpretability. It operates by recursively splitting the data into subsets based on feature values, forming a tree-like structure where each internal node represents a decision based on a specific feature, each branch represents an outcome of that decision, and each leaf node represents a class label. This hierarchical structure makes DT highly interpretable: one can easily trace the path from the root to a leaf to understand how a particular prediction was made, making it clear which features and thresholds are most influential. A key advantage of DT is its ability to model complex decision boundaries without requiring extensive data pre-processing. However, DT is prone to overfitting, especially when deep trees are used, as they may capture noise in the training data. Despite its limitations, DT remains a fundamental tool in machine learning research due to its intuitive nature and powerful performance in many applications.

RF is an ensemble learning method that enhances the performance of DTs by constructing a multitude of them during training and outputting the mode of the classes of the individual trees. This approach mitigates the overfitting typically associated with single DTs by introducing randomness through bootstrap sampling (bagging) and feature selection at each split. Each tree is built from a different subset of the data and considers only a random subset of features for splitting, promoting diversity among the trees and improving the overall model’s robustness. The aggregation of multiple trees results in a model that achieves high performance and generalizes well to new data. RF is highly versatile, capable of handling large datasets with high dimensionality, and providing insights into feature importance, which is valuable for understanding underlying data structures and making the model more interpretable. The method’s ability to rank features by their importance helps in identifying which variables have the most significant impact on predictions, making it easier to interpret the model’s decisions. Furthermore, RF can handle missing values effectively and maintain strong performance without extensive parameter tuning, adding to its practicality and popularity in machine learning research. Their ensemble nature ensures stability and resilience, making them suitable for a wide range of applications and data types.

### 3.3. Dataset

The dataset used in this experiment originates from the United Kingdom Adult ITP Registry, hosted jointly by QMUL and Barts Health NHS Trust. This registry, one of the largest international collections of adult primary ITP patients, encompasses detailed demographic, clinical, and genetic information from over 5000 individuals across more than 100 hospitals in the UK. With longitudinal follow-up data spanning several years, the registry serves as a comprehensive repository for understanding the characteristics and outcomes of primary ITP.

For this experiment, we utilised data from 150 patients; 100 primary ITP patients (from the UK Adult ITP Registry) and 50 non-ITP patients, selected from a non-acute general haematology outpatient clinic at Barts Health NHS Trust. In more detail, the dataset includes the following demographic features: age (ranging from 29 to 106 years old), gender (male and female), and race (Asian, Black (or African American), White (or Caucasian), and Other), along with key peripheral blood parameters at diagnosis: (i) blood alt (liver enzyme) level; (ii) blood haemoglobin level; (iii) blood neutrophil level; (iv) white blood cell count; (v) red blood cell count; (vi) blood platelet count; (vii) year of disease diagnosis. For our experiments, we employed a stratified five-fold cross-validation strategy.

**Figure 1:**
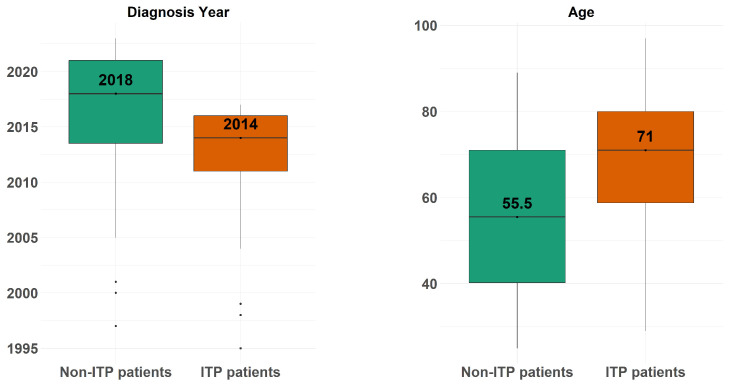
The boxplots of the patients’ ages (**right-hand side**) and year of disease diagnosis **(left-hand side**), across the ITP patients and non-ITP patients.

Figure 1 depicts the boxplots of the patients’ ages (right-hand side) and year of disease diagnosis (left-hand side), across the ITP patients and non-ITP patients.

**Figure 2:**
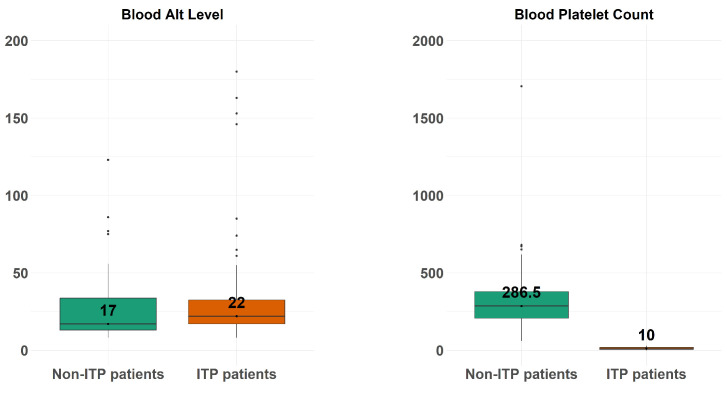
The boxplots of the patients’ blood platelet count (**right-hand side**) and blood alt level (**left-hand side**), across the ITP patients and non-ITP patients.

Figure 2 depicts the boxplots of the blood platelet count (right-hand side) and blood alt level (left-hand side) across the ITP patients and non-ITP patients.

**Figure 3:**
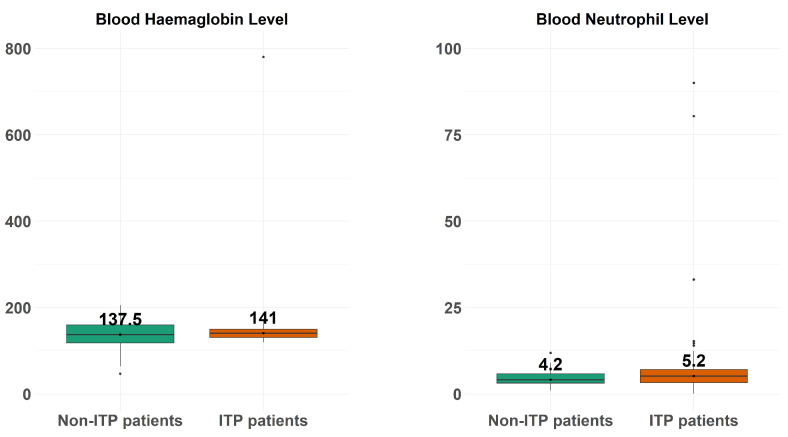
The boxplots of the blood neutrophil level (**right-hand side**) and blood haemoglobin level (**left-hand side**) across the ITP patients and non-ITP patients.

Figure 3 depicts the boxplots of the blood neutrophil level (right-hand side) and blood haemoglobin level (left-hand side) across the ITP patients and non-ITP patients.

**Figure 4:**
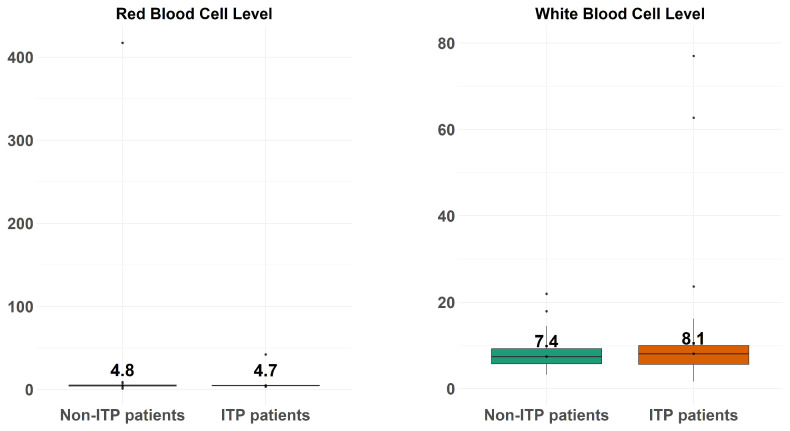
The boxplots of the white blood cell count (**right-hand side**) and red blood cell count (**left-hand side**) across the ITP patients and non-ITP patients.

Figure 4 depicts the boxplots of the white blood cell count (right-hand side) and red blood cell count (left-hand side) across the ITP patients and non-ITP patients.

**Figure 5:**
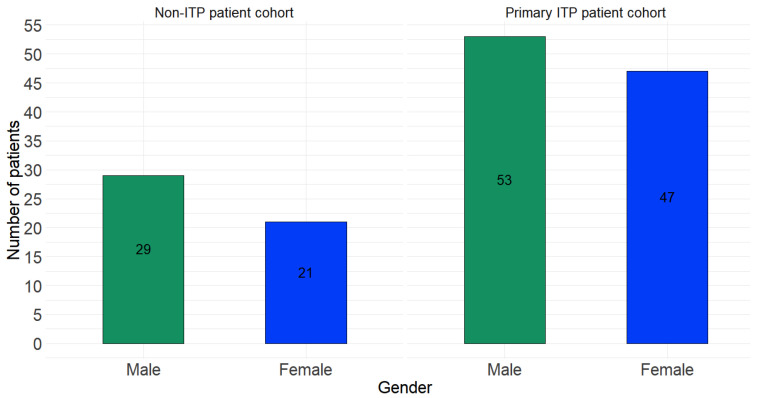
The gender distributions in the case of ITP (**right side**) and non-ITP (**left side**) patients.

Figure 5 presents the gender distributions (of male and female) in the case of ITP and non-ITP patients. One can see that in the ITP patient cohort, the percentage of males is 53%, and in the non-ITP cohort the corresponding percentage is 58% (i.e., 29 out of 50 patients). The distributions of male vs. female patients in each cohort are quite close (53–47% in the ITP cohort and 58–42% in the non-ITP cohort); the distributions of males in each cohort and the ones of females in each cohort are also quite similar. Finally, Table 1 presents all numeric variables that the previously described dataset contains, along with a small description of each, their minimum, maximum, median and mean values, as well as their reference/normal ranges.

### 3.4. Metrics

In the following, we present the metrics that we utilised for evaluating the performance of the ML models, as well as their fairness with respect to the sensitive variables: age, gender and race. Finally, we present the permutation feature importance technique that we utilised for measuring the importance of each individual variable in the models.

When performing a classification task, the most commonly used performance metric is the $F_{1}$ Score [18]. Generally speaking, the $F_{1}$ Score is a weighted average of the recall (i.e., the ability of the model classifier to find all the positive samples) and precision (i.e., the ability of the model classifier not to label as positive a sample that is negative). The $F_{1}$ Score takes values in the range [0,1]; high values are desired. The $F_{1}$ Score is defined as:

(1) $$F_{1}=\frac{2\times precision\times recall}{precision+recall}$$

In our case, the performance measure is the average $F_{1}$ Score (i.e., macro $F_{1}$ Score) across all two categories (i.e., ITP patient and non-ITP patient):

(2) $$P=\frac{F_{1}^{\mathrm{ITP}}+F_{1}^{\mathrm{NON}-\mathrm{ITP}}}{2}$$

However, the $F_{1}$ Score is not sufficient in exposing differences in performance (bias) in terms of the gender, age and ethnicity sensitive variables. Therefore, we also evaluate the models using a fairness metric. Fairness in ML is about ensuring that the models’ decisions do not favor or discriminate against particular groups based on sensitive attributes like race, gender and age. There are various definitions of fairness [19,20,21]. In this case, we use the Fairness of “Equalised Odds” [22].

Equalised Odds is a fairness metric whose goal is to ensure that a model’s performance is balanced in terms of both false positive rates (FPR) and true positive rates (TPR) across groups defined by sensitive attributes, such as race, gender, or age. TPR (also called sensitivity) is the probability that an actual positive will be correctly identified as positive. FPR is the probability that an actual negative will be wrongly identified as positive. Equalised Odds is achieved when a model satisfies the following condition: the probability of a positive prediction given the true label should be the same across different groups. This means that both the true positive rate (TPR) and false positive rate (FPR) should be equal across these groups. We define the Equalised Odds as the smaller of two metrics: true positive rate ratio and false positive rate ratio. The former is the ratio between the smallest and largest of $P[\bar{y}=1|y=1,s=\alpha ]$, across all values α of the sensitive variable *s*, with $\bar{y}$ being the model’s prediction (i.e., 0 or 1; in other words non-ITP or ITP patient), *y* being the target label (i.e., 0 or 1). For instance, for the sensitive variable ‘race’ the values are ‘White’, ‘Black’, ‘Asian’, ‘Other’. The latter is defined similarly, but for $P[\bar{y}=1|y=0,s=\alpha ]$. The Equalised Odds takes values in the range [0,1]; high values are desired (generally values of 90% or more indicate fair models); the Equalised Odds ratio of 1 means that all groups have the same true positive, true negative, false positive, and false negative rates.

Finally, we present the permutation feature importance technique [23] that we utilised for measuring the importance of each individual variable in the models. Permutation feature importance is a powerful tool for variable selection and model interpretation, helping in identifying variables that significantly impact the model’s predictive power, and those that do not contribute meaningfully and can potentially be removed without loss of performance. It is applicable to any model and is particularly useful because it is model agnostic—meaning it does not depend on the model internals and can be used with any ML model.

Permutation feature importance involves the following four steps. After a model is trained and evaluated, we select one variable in the dataset and permute (i.e., shuffle) its values among the data points. This disruption breaks the relationship between the variable and the target, effectively making the feature irrelevant. Next, with the permuted feature, we evaluate the model using the same performance metric that was used when it was originally trained. Because the association between the variable and the outcome has been disrupted, the model’s performance is expected to degrade if the variable was important. Following that, the importance of the variable is determined by the change in the model’s performance metric caused by shuffling the variable’s values. A significant decrease in performance indicates that the model relied heavily on that variable for making predictions. Conversely, a small or no change suggests that the variable was not very important for the model’s predictions. Finally, we perform this process for each variable in the dataset to gauge the relative importance of all features.

### 3.5. Pre-Processing and Implementation Details

In terms of pre-processing, we applied min-max normalisation [24] to each input variable independently. In terms of implementation details, we experiment with different hyperparameters for the ML models.

In the case of RF, we used 10 trees in the forest; in terms of the maximum depth of the tree, nodes were expanded until all leaves were pure (measured with regard to Gini impurity) or until all leaves contained one or no samples; one was the minimum number of samples required to be at a leaf node. In the case of DT, for the maximum depth of the tree, nodes were expanded until all leaves were pure (measured with regard to Gini impurity) or until all leaves contained one or no samples; one was the minimum number of samples required to be at a leaf node. In the case of LR, we added a constant (i.e., bias or intercept) to the decision function; we also added a L2 norm penalty term; we used the value of 1 as regularization parameter; the tolerance for stopping criterion used was 0.0001. In the case of SVM, the kernels that we have utilised in this work are the linear (LN), the radial basis function (RBF) and the polynomial with degree two, three and four (P2, P3 and P4, respectively). We used the value of 1 as regularization parameter; the penalty is the l2 norm and the loss function is the square of the hinge loss; the gamma value (only in the cases of RBF and polynomial kernels) is 1/number_of_features; the tolerance for stopping criterion used was 0.001. In the case of k-NN, we utilized 1, 2, 4, 8 and 12 nearest neighbours (in other words we used k = 1, 2, 4, 8 and 12); we used uniform weights and thus all points in each neighborhood were weighted equally; the metric used for the distance computation was the Euclidean distance. In the case of permutation feature importance, we selected 10 as the number of times to permute a variable. The scikit-learn library [25] was utilised for our implementations.

## 4. Experimental Results and Discussion

In the following we provide an extensive experimental study in which we compare the performance (in terms of the $F_{1}$ Score) of all utilised ML models, as well as the performance of the demographic-aware and the demographic-unaware approaches for each ML model. We also assess how biased each model is, by comparing their performance in terms of the Equalised Odds fairness metric, as well as how more or less biased the demographic-aware approach is to the demographic-unaware one. Finally, we present the permutation feature importance technique’s results for each ML model and for both approaches.

### 4.1. Demographic-Aware vs. Demographic-Unaware Performance Comparison across ML Models

Table 2 presents a performance comparison in terms of the $F_{1}$ Score (shown in %), across all folds, between the demographic-aware and demographic-unaware approaches of multiple machine learning methods (described in Section 3).

It can be observed that overall, the Random Forest (RF) method outperformed all others, achieving a 100% $F_{1}$ Score (i.e., perfect score) across all folds in both approaches, making it the most robust method. The second-best-performing method was the Decision Tree (DT) that also performed exceptionally well with near-perfect $F_{1}$ Scores. In particular, DT achieved an average $F_{1}$ across all folds of 99.2% in both approaches, achieving perfect (i.e., 100%) $F_{1}$ Scores on four out of five folds. One can also see in Table 2 that, for the k-NN method, best results have been achieved when the number of neighbors (k) was two, in both the demographic-aware and demographic-unaware approaches; overall, the higher the number of k considered, the worse the method’s performance was becoming in both approaches. Finally, for the SVM demographic-aware approach, PL kernels outperformed the standard RBF and LN kernels; moreover, the higher the order of the PL, the higher the degree of performance was achieved. The achieved results for the SVM demographic-unaware approach were more mixed (i.e., best performance was achieved when using a second order PL kernel; all PL kernels outperformed the LN kernel).

Finally, Table 2 shows that the demographic-unaware approach tends to perform better or equally across all machine learning models compared to the demographic-aware approach. Only the RF and DT methods achieved the same performance across the two approaches, which in turn means that they are more robust towards the inclusion or exclusion of demographic information; all other ML models achieved a better performance when demographic information was not included. When demographic information is included as input variables, models overfit these variables at the expense of others that are more generalisable. This degrades the performance since the demographic variables do not have a strong, consistent predictive relationship with the outcome. Moreover, demographic variables distract the model from focusing on more predictive variables, reducing its overall performance. The improvement in models without these features suggests that other variables might capture necessary information more effectively for the tasks at hand. The higher $F_{1}$ Scores observed in the demographic-unaware approach suggest that, in this specific context, excluding demographic information allows models to generalise better and focus on features that directly impact the outcome, leading to higher overall performance.

### 4.2. Demographic-Aware vs. Demographic-Unaware Fairness Comparison across ML Models

Table 3 presents a fairness comparison for the sensitive variables, gender, race and ethnicity, in terms of the Equalised Odds Score (presented in %), across all folds, between the demographic-aware and demographic-unaware approaches of multiple machine learning methods (described in Section 3). The following observations stand for all sensitive variables (gender, race and ethnicity).

It can be observed that overall, RF and DT models outperformed all others in the fairness metric; in other words, these models were the fairest among all ML methods. Furthermore, they were fair models as they achieved 100% and 90% Equalised Odds Scores, respectively (any model with ≥90% Equalised Odds Scores is considered fair). From Table 3, one can also note that RF and DT have identical fairness scores in both demographic-aware and demographic-unaware approaches, suggesting that these models’ fairness levels do not change with demographic information. It is worth noting that RF and DT achieved the best- and second-best performance in terms of $F_{1}$ Score, as can be seen in Table 2. Therefore, these models achieved both the best $F_{1}$ Score performance and the best Equalised Odds fairness performance. From Table 3, one can also notice that the rest of ML models are not fair as they achieved Equalised Odds Scores of ≤70%. These models did not also achieve the highest $F_{1}$ Score performance according to Table 2.

Table 3 further shows that, among the SVM models, SVM-LN is the fairest model and this observation is the same in both the demographic-aware and demographic-unaware approaches. For the polynomial SVM models, for the demographic-aware approach, one can see that the higher the degree of the polynomial, the fairer the performance is; whereas, for the demographic-unaware approach, the fairness score is the same no matter what the degree of the polynomial is. Additionally, Table 3 shows that, among the k-NN models, 2-NN is the fairest in the demographic-unaware approach and this model was also the best-performing in terms of $F_{1}$ Score (shown in Table 2). Generally, we see that, for the demographic-aware approach, the higher the k in k-NN (i.e., the more neighbors its has), the fairer the model is.

Finally, Table 3 shows that the demographic-aware approach tends to be fairer or equally fair (with regard to Equalised Odds Score), across all ML models, compared to the demographic-unaware approach. Only the RF and DT methods achieved the same fairness score across the two approaches (which means that they are more robust towards the inclusion or exclusion of demographic information); all other ML models achieved a higher fairness score when demographic information was included. This observation is the opposite from the one made when comparing their performance with regard to $F_{1}$ Score. In other words, demographic-aware approach is fairer but exhibits worse performance compared to demographic-unaware approach (RF and DT are the exception as these models achieved both the highest performance and highest fairness scores, whilst having same performance and fairness score between the demographic-aware and demographic-unaware approaches). This may seem contradictory or bizarre but in fact it is not.

In the demographic-aware approach, the sensitive variables were fed as input to the model which in turn paid particular attention to them and thought they were quite important and thus became fairer towards them. This is illustrated and proved in the next subsection that each ML model’s input feature importance is shown. However, in parallel, in the demographic-aware approach, the models achieved worse performance.

The existence of a trade-off between model performance and fairness is known in the ML community [26,27,28] which is primarily due to inherent differences in data distributions, historical biases present in the training data, and the conflicting goals of optimising for performance and accuracy versus equity. Groups within a dataset might exhibit different statistical patterns due to a variety of factors. When a model aims for high performance, it leverages these patterns to make predictions. However, fairness considerations often require the model to treat different groups similarly, even if their underlying distributions differ. Adjusting the model to ignore these differences (to achieve fairness) can lead to a reduction in performance because the model is no longer fully optimised according to the natural distributions of the data. Such adjustments can force the model to choose less optimal solutions from a purely statistical perspective in order to enhance equity. This approach might mean rejecting the statistically “best” model in favor of one that is less accurate overall but more balanced in terms of demographic impact.

### 4.3. Feature Importance of Demographic-Aware vs. Demographic-Unaware ML Models

**Figure 6:**
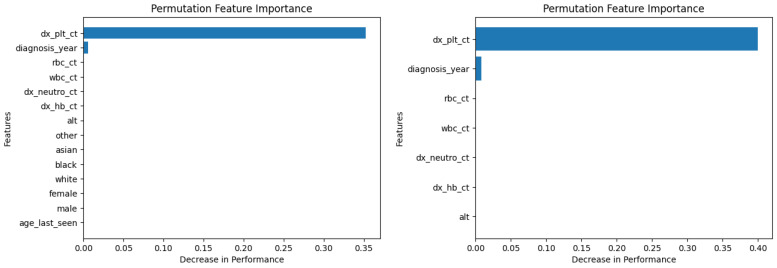
Permutation feature importance on the training set for the RF model in the case of the demographic-aware (on the **left side**) and demographic-unaware (on the **right side**) approach.

**Figure 7:**
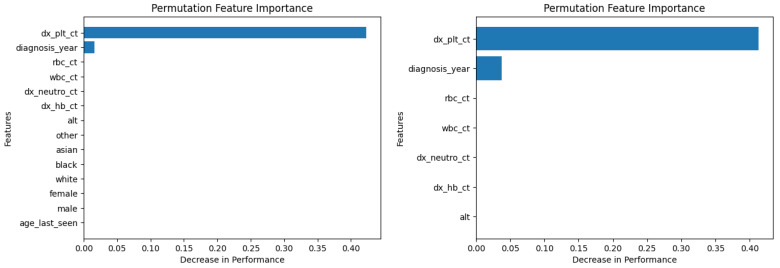
Permutation feature importance on the test set for the RF model in the case of the demographic-aware (on the **left side**) and demographic-unaware (on the **right side**) approach.

**Figure 8:**
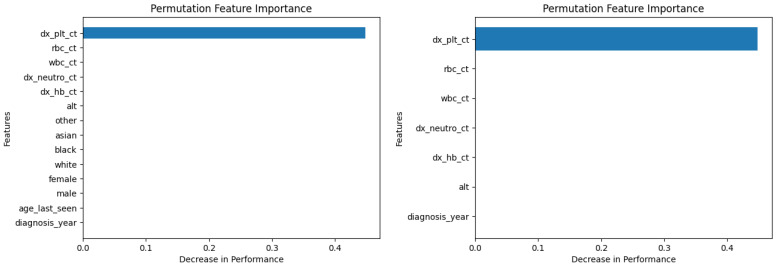
Permutation feature importance on the training set for the DT model in the case of the demographic-aware (on the **left side**) and demographic-unaware (on the **right side**) approach.

**Figure 9:**
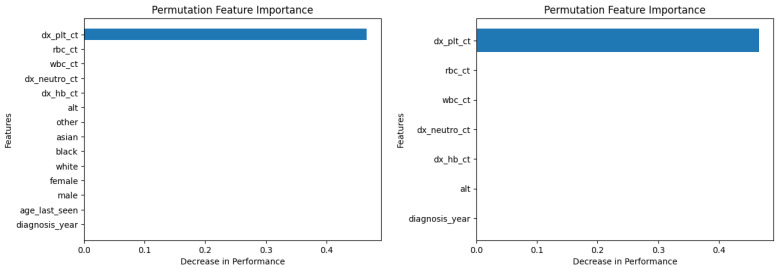
Permutation feature importance on the test set for the DT model in the case of the demographic-aware (on the **left side**) and demographic-unaware (on the **right side**) approach.

Figure 6 and Figure 7 show the permutation feature importance on the training and test sets, respectively, for the RF model in the case of the demographic-aware (on the left side) and demographic-unaware (on the right side) approach. Let us note that there were similar distributions and findings across all training and test sets of each fold (of the five-fold cross validation); this was the case for all ML methods that we present in this subsection. Figure 8 and Figure 9 illustrate the same information for the DT model. In all these Figures and cases, one can see that the most important and influential input variable for the ML model’s decision is ‘dx_plt_ct’, i.e., the blood platelet count, regardless if we are examining the training or the test set. This is consistent with medical findings; medical experts mainly diagnose ITP based on the platelet count. Let us note that these two methods (RF and DT) are the ones that: (i) achieved the best (and perfect or almost perfect) performance in diagnosing ITP (according to Table 2) compared to the other ML models; (ii) are fair in terms of the sensitive variables (age, gender and race), as well as are the fairest among all ML models (according to Table 3); (iii) achieved the same performance and fairness in the demographic-aware and demographic-unaware approach.

**Figure 10:**
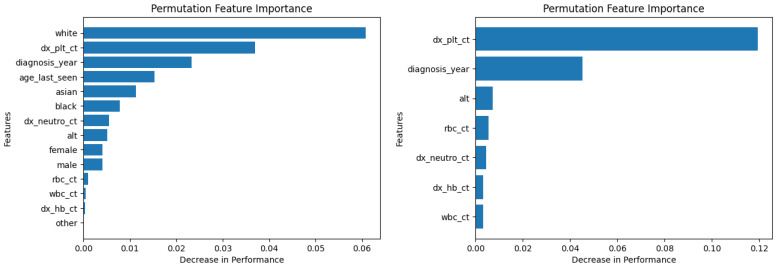
Permutation feature importance on the training set for the LogR model in the case of the demographic-aware (on the **left side**) and demographic-unaware (on the **right side**) approach.

**Figure 11:**
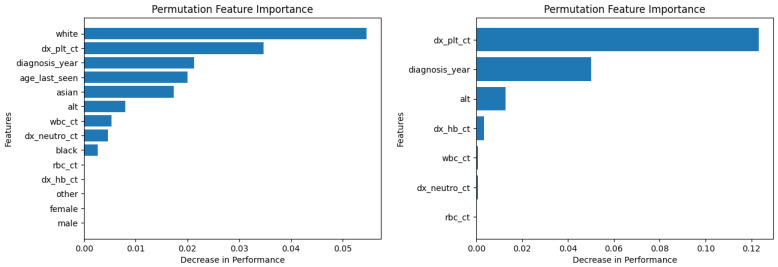
Permutation feature importance on the test set for the LogR model in the case of the demographic-aware (on the **left side**) and demographic-unaware (on the **right side**) approach.

**Figure 12:**
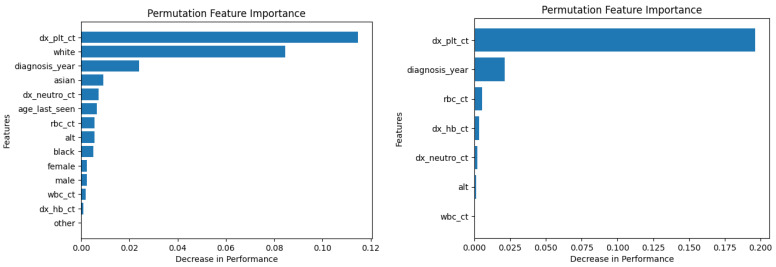
Permutation feature importance on the training set for the SVM-LN model in the case of the demographic-aware (on the **left side**) and demographic-unaware (on the **right side**) approach.

**Figure 13:**
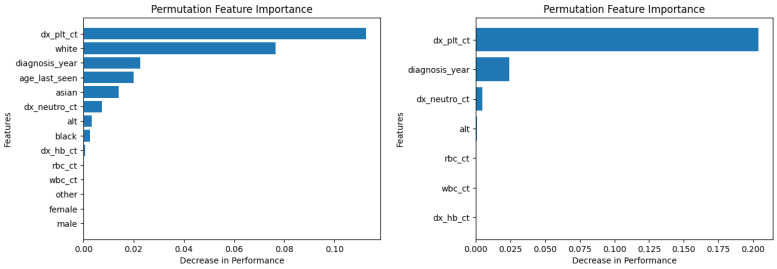
Permutation feature importance on the test set for the SVM-LN model in the case of the demographic-aware (on the **left side**) and demographic-unaware (on the **right side**) approach.

**Figure 14:**
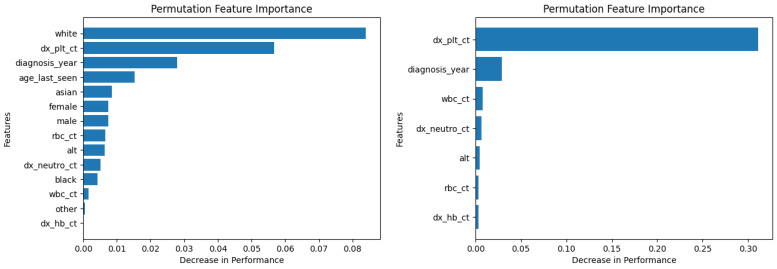
Permutation feature importance on the training set for the SVM-RBF model in the case of the demographic-aware (on the **left side**) and demographic-unaware (on the **right side**) approach.

**Figure 15:**
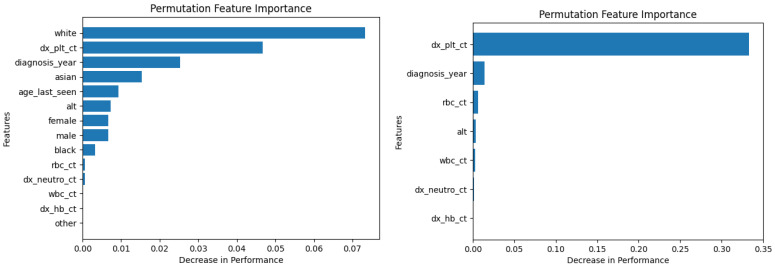
Permutation feature importance on the test set for the SVM-RBF model in the case of the demographic-aware (on the **left side**) and demographic-unaware (on the **right side**) approach.

**Figure 16:**
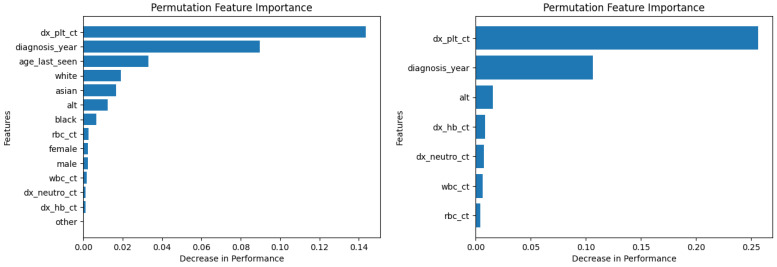
Permutation feature importance on the training set for the 2-NN model in the case of the demographic-aware (on the **left side**) and demographic-unaware (on the **right side**) approach.

**Figure 17:**
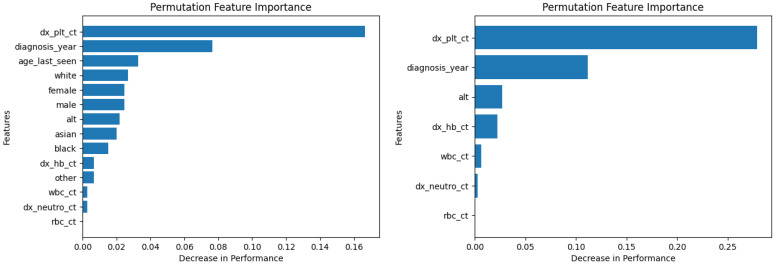
Permutation feature importance on the test set for the 2-NN model in the case of the demographic-aware (on the **left side**) and demographic-unaware (on the **right side**) approach.

**Figure 18:**
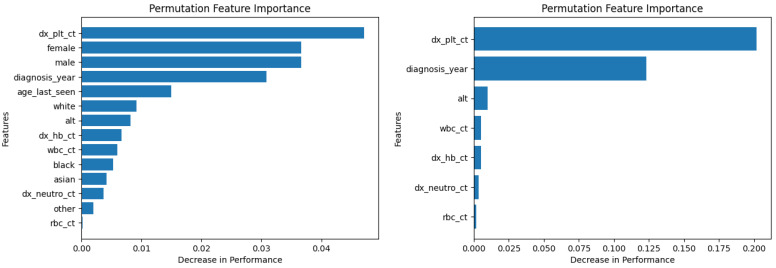
Permutation feature importance on the training set for the 12-NN model in the case of the demographic-aware (on the **left side**) and demographic-unaware (on the **right side**) approach.

**Figure 19:**
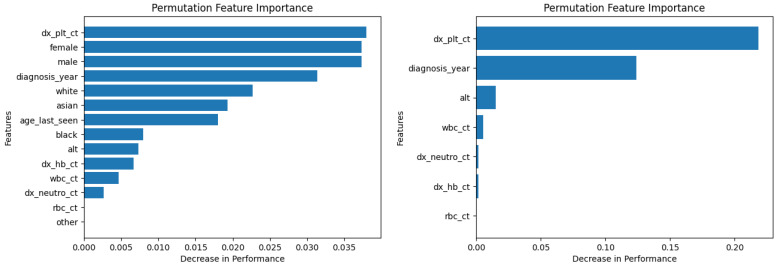
Permutation feature importance on the test set for the 12-NN model in the case of the demographic-aware (on the **left side**) and demographic-unaware (on the **right side**) approach.

Similarly, Figure 10, Figure 11, Figure 12, Figure 13, Figure 14, Figure 15, Figure 16, Figure 17, Figure 18 and Figure 19 show the permutation feature importance on the training set (and test set) for the LogR, SVM-LN, SVM-RBF, 2-NN, and 12-NN models, respectively, in the case of the demographic-aware (on the left side) and demographic-unaware (on the right side) approach. In all cases, one can see that ‘dx_plt_ct’ is in the top-two most important variables.

These Figures prove that, for the demographic-aware approach, the sensitive variables play an important role in the models’ decisions. In all ML models, mainly the race and age are always in the top-two most important input variables that cause the most significant decrease in performance. They also show that, for all ML models, the input variable ‘dx_plt_ct’ (i.e., the blood platelet count) plays a more important role in the demographic-unaware approach compared to the demographic-aware one; in the case of the demographic-unaware approach, the decrease in model’s performance is between two and seven times bigger than the corresponding one of the demographic-aware approach (for instance, for SVM-RBF, for ‘dx_plt_ct’, the decrease in performance is around 0.06 in the demographic-aware approach and 0.30 in the demographic-unaware approach).

Let us also note that from Table 2 12-NN (in the demographic-aware approach) and LogR (in both approaches) were the worst-performing models. This is explained in Figure 10, Figure 11, Figure 18 and Figure 19 as ‘dx_plt_ct’ exhibits the smallest decrease in performance (around 0.04 for the demographic-aware and 0.12 for the demographic-unaware approach) among all ML models.

## 5. Conclusions

In conclusion, this feasibility study demonstrates the potential of ML models to significantly enhance the diagnostic process for Primary Immune Thrombocytopenia (ITP) in non-acute outpatient settings. By analysing routine blood tests and demographic information, models such as the Random Forest and Decision Tree were found to provide high (and the highest among all utilized ML models) predictive performance and fairness, performing robustly across different subsets of data. The results for the remaining ML models indicate that, while models that are not presented with demographic information often achieved higher predictive performance, those presented with demographic information showed higher fairness, highlighting the complex balance between model performance and fairness. Only the Random Forest and Decision Tree models achieved the same predictive performance and fairness when presented vs. when not presented with demographic information; this result indicates that demographic information is not important for the (actual) diagnosis of ITP and thus should not be used as an input variable. In that way, there will be no ethical implications nor privacy concerns when deploying such a system for clinical usage, as the data will be anonymized and de-identifiable. Importantly, this study identified platelet count as the most critical predictor of ITP, confirming the relevance of this parameter in clinical diagnostics. By facilitating earlier and more accurate diagnosis, the implementation of such ML models could lead to better patient management and potentially reduce the healthcare system burden associated with ITP.

Finally, let us mention that this work was an initial attempt—a feasibility study—to check if ML models can achieve effective and fair diagnosis of ITP using routine blood tests and demographic data in a non-acute outpatient setting. That is why the utilized dataset is not particularly large (although it is adequate for demonstrating the potential of using ML for this purpose); for clinical usage, a larger and more diverse dataset is needed for more robust model development. Therefore, our future plans include, at first, expanding this study by incorporating a bigger data corpus (bigger in terms of the total number of subjects, total number of ITP and non-ITP patients, more input variables, more conditions and causes of ITP, as well as more diseases in the non-ITP cases), and then developing an effective, efficient, fair and explainable ML model. We will further propose a strategy to mitigate bias in the model. After such a model is developed, we will conduct a pre-clinical study with human participants in real life and in real-world conditions as the final test before the model is put to clinical usage within the UK Adult ITP Registry.

## Figures and Tables

**Table 1:** Data statistics for each numeric variable of the utilised datasets in this work. The Table also contains a small description of each variable, as well as their reference/normal ranges.

		Primary ITP Patients				Non-Primary ITP Patients				
Variable Name	Description	Min	Max	Median	Mean	Min	Max	Median	Mean	Reference Ranges
diagnosis_year	Disease diagnosis year	1995	2017	-	-	1997	2023	-	-	-
age_last_seen	Age last seen in clinic	29	106	71	67	25	89	55.5	55.9	-
alt	Blood ALT value	8	180	22	31.7	8	123	17	27.2	7–40
dx_hb_ct	Blood haemoglobin count	120	780	141	148	47	206	137.5	139	115–180
dx_neutro_ct	Blood neutrophil count	0.17	90	5.2	7.4	1.1	11.9	4.15	4.15	1.5–8.0
wbc_ct	White Blood Cell count	1.6	77	8.05	9.5	3.2	21.9	7.4	8	4.0–11.0
rbc_ct	Red Blood Cell count	3.3	42	4.71	5.14	1.09	417	4.81	13	3.66–5.54
dx_plt_ct	Blood Platelet count	0	29	10	12.3	60	1705	286.5	340	150–450

**Table 2:** $F_{1}$ Score performance comparison (in %) between the demographic-aware and demographic-unaware approaches across Logistic Regression (LogR), Support Vector Machines (SVM) with different kernels (radial basis function, linear and polynomial of various orders), k-Nearest Neighbors (with different ‘k’), Decision Tree (DT) and Random Forest (RF). High $F_{1}$ Score values are desired. Best scores are in bold; second best scores are underlined; best scores of each ML model are in italics.

Method	$F_{1}$ Score											
	Demographic-Aware Approach						Demographic-Unaware Approach					
	Fld 1	Fld 2	Fld 3	Fld 4	Fld 5	Mean	Fld 1	Fld 2	Fld 3	Fld 4	Fld 5	Mean
LogR	72.1	65.9	77.8	68.9	79.5	72.8	75.1	61.3	80.8	75.1	75.1	73.5
SVM-RBF	72.1	65.9	72.1	74.4	79.5	72.8	87.7	92.1	87.7	88.5	96.2	90.4
SVM-LN	83	65.9	87.7	84.1	84.1	81	77.1	77.1	82.8	88	88	82.6
SVM-P2	83	71.3	83	84.1	88.5	82	*83*	*96.2*	*87.7*	*92.1*	*96.2*	*91*
SVM-P3	83	85	83	96.3	96.3	88.7	83.5	88.2	88.1	88.2	96.7	88.9
*SVM-P4*	*83*	*89*	*83*	*92.8*	*96.3*	*88.8*	83	87.7	92.1	87.7	96.2	89.3
1-NN	83	85	83	82.4	89	84.5	96.2	100	92.8	89	96.2	94.8
*2-NN*	*83*	*89*	*92.1*	*79.2*	*85.7*	*85.8*	*96.2*	*96.2*	*96.2*	*92.5*	*96.2*	*95.4*
4-NN	83	80.8	87.7	84.1	88.5	84.8	87.7	92.1	96.2	84.1	96.2	91.2
8-NN	77.8	83	83	71.3	76.2	78.2	92.1	92.1	96.2	79.5	96.2	91
12-NN	65.6	58.3	72.1	62.7	74.3	66.6	83	92.1	87.7	87.7	92.1	88.5
DT	100	96.2	100	100	100	99.2	100	96.2	100	100	100	99.2
**RF**	**100**	**100**	**100**	**100**	**100**	**100**	**100**	**100**	**100**	**100**	**100**	**100**

**Table 3:** Sensitive variables (gender, race, age): Fairness comparison in terms of Equalised Odds (in %) between the demographic-aware and demographic-unaware approaches across Logistic Regression (LogR), Support Vector Machines (SVM) with different kernels (radial basis function, linear and polynomial of various orders), k-Nearest Neighbors (with different ‘k’), Decision Tree (DT) and Random Forest (RF). High values of Equalized Odds are desired (generally values of 90% or more indicate fair models). Best scores are in bold; second best scores are underlined; best scores of each ML model are in italics.

Method	Equalised Odds Score					
	Demographic-Aware Approach			Demographic-Unaware Approach		
	Gender	Race	Age	Gender	Race	Age
LogR	61.1	52.2	47.9	49.8	43.1	37.5
SVM-RBF	52.1	48.1	45	30	37.9	39
SVM-LN	*62.2*	*54.6*	*62.3*	*43.6*	*54.1*	*57.4*
SVM-P2	34.6	36.1	40.4	22.4	26.3	28
SVM-P3	45.8	52.1	55.1	22.4	26.3	28
SVM-P4	46.2	52.8	45.7	22.4	26.3	28
1-NN	20.1	21.2	24	12.2	13.6	15.2
2-NN	36	33.4	29.5	*31.6*	*32.4*	*29.1*
4-NN	42	38.9	33.3	28.4	31.5	28.2
8-NN	56.7	48.6	37.5	19.7	26.7	27.1
12-NN	*69.6*	*55.4*	*42.4*	16.4	18.9	23.8
DT	90	90	90	90	90	90
**RF**	**100**	**100**	**100**	**100**	**100**	**100**

## Data Availability

Data can be available upon request from the corresponding authors.

## References

[1] Provan D., Semple J. (2022). Recent advances in the mechanisms and treatment of immune thrombocytopenia. EBioMedicine.

[2] Doobaree I.U., Conway K., Miah H., Miah A., Makris M., Hill Q., Cooper N., Bradbury C., Newland A., Provan D. (2022). Incidence of adult primary immune thrombocytopenia in England—An update. Eur. J. Haematol..

[3] Kollias D., Tagaris A., Stafylopatis A., Kollias S., Tagaris G. (2018). Deep neural architectures for prediction in healthcare. Complex Intell. Syst..

[4] Malik P., Pathania M., Rathaur V.K. (2019). Overview of artificial intelligence in medicine. J. Fam. Med. Prim. Care.

[5] Mani V., Ghonge M.M., Chaitanya N.K., Pal O., Sharma M., Mohan S., Ahmadian A. (2022). A new blockchain and fog computing model for blood pressure medical sensor data storage. Comput. Electr. Eng..

[6] Chowdhury D., Das A., Dey A., Banerjee S., Golec M., Kollias D., Kumar M., Kaur G., Kaur R., Arya R.C. (2023). CoviDetector: A transfer learning-based semi supervised approach to detect Covid-19 using CXR images. Benchcouncil Trans. Benchmarks Stand. Eval..

[7] Zhao C., Xiang S., Wang Y., Cai Z., Shen J., Zhou S., Zhao D., Su W., Guo S., Li S. (2023). Context-aware network fusing transformer and V-Net for semi-supervised segmentation of 3D left atrium. Expert Syst. Appl..

[8] Iwendi C., Huescas C., Chakraborty C., Mohan S. (2024). COVID-19 health analysis and prediction using machine learning algorithms for Mexico and Brazil patients. J. Exp. Theor. Artif. Intell..

[9] An Z.Y., Wu Y.J., Hou Y., Mei H., Nong W.X., Li W.Q., Zhou H., Feng R., Shen J.P., Peng J. (2023). A life-threatening bleeding prediction model for immune thrombocytopenia based on personalized machine learning: A nationwide prospective cohort study. Sci. Bull..

[10] Chong S., Zhao P., Huang R.B., Zhou H., Zhang J.N., Hou M., Liu Y., Yao H.X., Niu T., Peng J. (2022). Developing and validating a mortality prediction model for ICH in ITP: A nationwide representative multicenter study. Blood Adv..

[11] Kim T.O., MacMath D., Pettit R.W., Kirk S.E., Grimes A.B., Gilbert M.M., Powers J.M., Despotovic J.M. (2021). Predicting Chronic Immune Thrombocytopenia in Pediatric Patients at Disease Presentation: Leveraging Clinical and Laboratory Characteristics Via Machine Learning Models. Blood.

[12] Liu F.Q., Chen Q., Qu Q., Sun X., Huang Q.S., He Y., Zhu X., Wang C., Fu H.X., Li Y.Y. (2021). Machine-Learning Model for Resistance/Relapse Prediction in Immune Thrombocytopenia Using Gut Microbiota and Function Signatures. Blood.

[13] Menard S. (2002). Applied Logistic Regression Analysis.

[14] Hearst M.A., Dumais S.T., Osuna E., Platt J., Scholkopf B. (1998). Support vector machines. IEEE Intell. Syst. Their Appl..

[15] Peterson L.E. (2009). K-nearest neighbor. Scholarpedia.

[16] Song Y.Y., Ying L. (2015). Decision tree methods: Applications for classification and prediction. Shanghai Arch. Psychiatry.

[17] Breiman L. (2001). Random forests. Mach. Learn..

[18] Grandini M., Bagli E., Visani G. (2020). Metrics for multi-class classification: An overview. arXiv.

[19] Verma S., Rubin J. Fairness definitions explained. Proceedings of the International Workshop on Software Fairness.

[20] Mehrabi N., Morstatter F., Saxena N., Lerman K., Galstyan A. (2021). A survey on bias and fairness in machine learning. ACM Comput. Surv. (CSUR).

[21] Barocas S., Hardt M., Narayanan A. (2023). Fairness and Machine Learning: Limitations and Opportunities.

[22] Garg P., Villasenor J., Foggo V. (2020). Fairness metrics: A comparative analysis. Proceedings of the 2020 IEEE International Conference on Big Data (Big Data).

[23] Altmann A., Toloşi L., Sander O., Lengauer T. (2010). Permutation importance: A corrected feature importance measure. Bioinformatics.

[24] Singh D., Singh B. (2020). Investigating the impact of data normalization on classification performance. Appl. Soft Comput..

[25] Pedregosa F., Varoquaux G., Gramfort A., Michel V., Thirion B., Grisel O., Blondel M., Prettenhofer P., Weiss R., Dubourg V. (2011). Scikit-learn: Machine learning in Python. J. Mach. Learn. Res..

[26] Kleinberg J., Mullainathan S., Raghavan M. (2016). Inherent trade-offs in the fair determination of risk scores. arXiv.

[27] Ma X., Wang Z., Liu W. (2022). On the tradeoff between robustness and fairness. Adv. Neural Inf. Process. Syst..

[28] Menon A.K., Williamson R.C. The cost of fairness in binary classification. Proceedings of the Conference on Fairness, Accountability and Transparency.

